# When parents face the death of their child: a nationwide cross-sectional survey of parental perspectives on their child’s end-of life care

**DOI:** 10.1186/s12904-016-0098-3

**Published:** 2016-03-09

**Authors:** Karin Zimmermann, Eva Bergstraesser, Sandra Engberg, Anne-Sylvie Ramelet, Katrin Marfurt-Russenberger, Nicolas Von der Weid, Chantal Grandjean, Patricia Fahrni-Nater, Eva Cignacco, on behalf of the PELICAN Consortium

**Affiliations:** Institute of Nursing Science, University of Basel, Bernoullistrasse 28, 4056 Basel, Switzerland; Department of Paediatrics, Inselspital Bern University Hospital, Bern, Switzerland; Paediatric Palliative Care, University Children’s Hospital Zurich, Steinwiesstrasse 75, 8032 Zurich, Switzerland; School of Nursing, University of Pittsburgh, 3500 Victoria Street, Pittsburgh, PA 15261 USA; Institute of Higher Education and Research in Healthcare - IUFRS, University of Lausanne, Route de la Corniche 10, 1010 Lausanne, Switzerland; Paediatric Department, Lausanne University Hospital CHUV, Rue du Bugnon 21, 1011 Lausanne, Switzerland; Department of Paediatric Haematology-Oncology, University Children’s Hospital UKBB, Spitalstrasse 33, 4056 Basel, Switzerland; Paediatric Palliative Care Team, Lausanne University Hospital CHUV, Rue du Bugnon 21, 1011 Lausanne, Switzerland; Research in Midwifery, University of Applied Sciences Bern, Health Division, Bern, Switzerland

**Keywords:** Paediatrics, End-of-life, Terminal care, Questionnaire survey, Parental perspectives

## Abstract

**Background:**

Parents facing the death of their child have a strong need for compassionate professional support. Care services should be based on empirical evidence, be sensitive to the needs of the families concerned, take into account the heterogeneity within the medical field of paediatrics, and fit into the local health care system. We need to better understand the perspectives of parents facing the death of their child in order to guide further development and evaluation of specialised paediatric palliative and end-of-life (EOL) care services.

**Methods:**

Questionnaire survey to assess the EOL care perspectives of a Swiss population-based sample of bereaved parents who had lost a child due to a cardiac, neurological or oncological condition, or during the neonatal period in the years 2011 or 2012. The parental perspective was assessed with a newly developed and tested instrument that was structured according to six evidence-based quality domains. Responses regarding parental experiences and perceived satisfaction are described. Differences between the four diagnostic groups are analysed using a generalized estimation equation to account for the dyadic data structure.

**Results:**

Of 307 eligible families, 267 could be contacted and 135 (51 %) consented to participate in this questionnaire survey. Our findings show positive parental experiences of their child’s EOL care and high perceived satisfaction with the care their child received. Parents of a child with cancer rated their experiences highest in most of the six quality domains and reported the highest satisfaction with care. The lowest scores were mainly reported by parents from the neurology group, with the exception of the shared decision making domain, where parents of neonates reported significantly less positive experiences.

**Conclusions:**

Although positive in general, our study results suggest some areas for improvement. The integration of specialised paediatric palliative care has the potential to minimise lost opportunities to support and assist parents.

**Electronic supplementary material:**

The online version of this article (doi:10.1186/s12904-016-0098-3) contains supplementary material, which is available to authorized users.

## Background

When facing the death of their child, parents experience an unimaginably painful life event and severe crisis that affects the whole family for life. In this highly stressful time parents are confronted with uncertainty and are required to make difficult decisions, e.g. withdrawal of life-sustaining interventions. Their need for compassionate professional support is high. Support throughout terminal care and after the loss of a child was reported to have a positive impact on long-term grieving outcomes of parents who lost a child to cancer [1]. For clinicians it is therefore imperative to know how parents experience their child’s end-of-life (EOL) and what their specific needs are in order to provide good quality care. Two recent integrative reviews and a qualitative metasummary extracted existing evidence from 36 studies (29 qualitative, 7 quantitative) about parental perspectives on their child’s palliative care (PC) or EOL care [2–4]. This evidence provides an overview of themes/domains most important to parents and can be summarised as: sincere relationships and emotional, spiritual and cultural support; genuine communication; alleviation of suffering; continuity, coordination and accessibility of care; and bereavement support [2–4]. Deficiencies in meeting parental needs were identified across all themes, e.g. insufficient communication, lack of respect, and lack of emotional support [2].

Caring for a child at the end of her/his life and supporting the family is most challenging for health care professionals. It requires a skilled multidisciplinary health care team that adopts a comprehensive and integrative care approach [5]. This has led to the emergence of the medical subspecialty of paediatric palliative care (PPC), which is defined by the World Health Organization as the active total care of the child’s body, mind and spirit, and involves giving support to the family. It requires a broad approach that includes the family. It can be provided in tertiary care facilities, in community health centres and even in children’s homes [6]. Many countries have recognized the need for PPC and a series of hospital-based programs have been developed and implemented during the last decade [7, 8]. In Switzerland, this need is acknowledged by the Federal Office of Public Health by incorporating it in its national strategy and conception for implementation [9, 10]. Their proposed framework emphasizes the importance of a person-centred approach focusing on the complexity of the situation and needs of the person concerned [10]. In the field of paediatrics especially, person-centeredness must be extended to family-centredness, with the child and family as the unit of care. Paediatric care encompasses the whole age continuum from infants and children who have never experienced or expressed preferences to adolescents able to discuss their situation and express expectations [11], and it takes place in various in- and outpatient care settings and at home.

Fortunately, childhood deaths are a rare event. In Switzerland 424 deaths in children (0 to 14 years of age) were registered in 2013. Mortality data from developed countries show, that perinatal conditions contribute to 50 % of all deaths in the first year of life. Beyond the first year, external causes, e.g. accidents, are the most common causes of death. Complex chronic conditions such as genetic/congenital disorders, neurological and cardiac conditions, and cancer represent the main causes of disease-related deaths [12]. This wide variety of underlying medical conditions leads to vastly different illness trajectories and lifespans potentially influencing what parents experience during their child’s EOL care. There is little evidence concerning the influence of the child’s underlying diagnosis on the parental perspective. It has been suggested that different challenges arise and that families from the oncology group can generally draw on a better developed professional support infrastructure than other affected families [13, 14].

In many of the existing studies covering parental perspectives of their child’s PPC or EOL care, samples were limited either by case numbers, the inclusion of underlying illnesses causing the child’s death (i.e. predominantly parents of children with cancer [13]) or the care setting (e.g. paediatric intensive care unit) [15, 16]. Care services should be based on empirical evidence, sensitive to the needs of the families concerned, take into account the heterogeneity within the medical field of paediatrics, and should fit the local health care system. We need to better understand the perspectives of parents facing the death of their child in order to guide further development and evaluation of specialised PPC and EOL care services. It was therefore the purpose of this study to assess the perspectives of bereaved parents who had lost a child due to a cardiac, neurological or oncological condition, or during the neonatal period in order to (1) describe specific parental experiences in relation to the underlying medical condition causing the child’s death, and (2) explore differences in parental perspectives between four common medical conditions responsible for childhood death.

## Methods

### Design, setting, participants, and recruitment

The cross-sectional questionnaire survey was embedded in a larger research project concerned with paediatric EOL care needs in Switzerland (Paediatric End-of-LIfe CAre Needs – PELICAN, 2012-2015, NCT01983852) drawing from a population based sample of deceased children, their bereaved parents and health care professionals. The PELICAN study aimed to provide comprehensive information and understanding about the current practices of EOL care (in this study, defined as the last 4 weeks of life prior to death) in paediatric settings in Switzerland (hospital and community care), and about the perspectives of the parents and health care professionals involved [17]. The questionnaire survey reported on here covered the quantitative assessment of parental perspectives by including parents of all children that had died due to a cardiac, neurological or oncological condition or during the neonatal period in the years 2011 and 2012. These four groups were chosen, as they represent the major diagnoses causing illness-related death in children [18]. Eligible parents were identified using administrative death data from all Swiss children’s hospitals, general hospitals with a paediatric unit, long-term institutions and paediatric community care services. All institutions with probable events of death were informed of the study and committed to participate and execute the recruitment procedures, which involved sending out an invitation letter together with the informed consent documents. Parents were not invited if their child had died during the first 24 h of her/his life. Parents were included if they consented to participate and were proficient in the German, French or Italian language. Once parents sent back their written consent, their demographic information was then transmitted to the research team. If written consent was not received three weeks after receipt of the study documents, the family was telephoned by a local study coordinator to provide verbal study information and to clarify potential questions. Recruitment occurred between July 2013 and March 2014 in 8 children’s hospitals (5 of them tertiary paediatric care centres), 9 general hospitals with a paediatric unit, 2 long-term institutions, and 4 paediatric community care services. For two families, the hospital delegated recruitment to a paediatric practitioner’s practice which then invited the family. Human Research Ethics Committees from the 11 Swiss cantons in which the recruiting institutions were located approved the PELICAN study (leading committee: KEK ZH Nr. 2012-0537, Additional file 1) [19].

### Measures

To retrospectively assess the parental perspective on the child’s EOL care, a survey instrument, the Parental PELICAN Questionnaire (PaPEQu) was developed by the PELICAN study group. A detailed description of the development and validation of the PaPEQu has been published elsewhere [19], and a complete list of items with corresponding response options is provided (Additional file 2). Initial validity and reliability of the PaPEQu were demonstrated in a sample of health care professionals and bereaved parents [19].

Four slightly different versions for the four diagnostic groups (cardiology, neonatology, neurology, and oncology) were created to account for differences in illness trajectories between the groups. The PaPEQU is thematically structured following the framework of six quality domains identified by the Initiative for Pediatric Palliative Care [20] and adapted by Truog et al. [21]. The six domains are in accordance with existing evidence and include: *support of the family unit, communication, shared decision making, relief of pain and other symptoms, continuity of care, and bereavement support*. Within each domain, the items were organised into scales or single items related to parental experiences and indexes for parental needs. The item count of experience related items ranged from 44 to 48 items, depending on the diagnostic group version. With 34 needs-related items and 13 socio-demographic items, the total item count of the PaPEQu ranged from 91 to 95 items. In this article, we report on the items related to parental experiences and socio-demographic information only. The results of the needs-related items showed high ceiling effects and little variation across the four diagnostic groups, making a thorough interpretation of these results difficult. Those items can still be checked in the complete item list (Additional file 2).

For experience-related scale items, the response option was either a 7-point (0 to 6) with varying end-point anchors (*“never-always”*, *“not clear at all-very clear”*, *“not honest-honest”)*, or a 5-point Likert-type (1 to 5), where respondents indicated the extent to which they agreed with the statement. The assessment of parental experiences was supplemented with single items with multiple choice or dichotomous response options (Yes-No) as appropriate. In addition, parents were asked to rate their perceived overall satisfaction with the care their child received for each of the six quality domains on a 7-point scale (1 to 7) with end-point anchors *“not satisfied at all -satisfied”* and a *“neutral”* label in the middle. They were also asked to list three positive and three negative experiences associated with their child’s EOL care and to indicate what areas of their lives were negatively influenced by their child’s illness and death with a question allowing multiple responses. Finally, on a 0 (worst possible) to 10 (best possible) vertical visual analogue scale they were asked to rate their current quality of life (QoL). Socio-demographic information was collected at the end of the questionnaire. Scale items were summed and averaged to yield one score per domain with higher values representing more positive experiences. The unidimensionality of the parental experiences score for each domain separately was demonstrated with exploratory factor analysis; internal consistency testing showed Cronbach’s alpha levels between 0.69–0.88 [19].

### Study procedures

The PaPEQu was sent out in April 2014 to mothers and fathers who individually consented to participate in this part of the PELICAN study. An identification code was assigned to each questionnaire allowing mapping of the family dyad. Parents who had not sent back the completed questionnaire within three weeks received a reminder card. Non-responders to this reminder were dropped from the study. All questionnaires were hand checked for completeness upon receipt, and electronically scanned to be downloaded onto an IBM® SPSS® data file.

### Data analysis

The responses to all items were explored using measures of central tendency and dispersion. Descriptive statistics were used to summarize parental experiences for the total sample as well as for the four diagnostic groups. For each item the percentage and pattern of missing responses were calculated and explored using missing value analysis. Items that more than 30 % of respondents either did not answer or responded to with “not applicable”/“don’t know” were only analysed descriptively.

Scale items related to parental experiences that were present in all four questionnaire versions were used to calculate a scale score as described above. All other items were analysed as single items. Since most responses were skewed showing a ceiling effect, data transformation was applied. i.e. base 10 logarithm, square root, or reciprocal as indicated [22], for continuous dependent variables. Correction of severe distributional violations such as the presence of outliers was achieved. To explore differences in parental perspectives between the four diagnostic groups, various statistical tests were applied. For the experiences scores and the perceived satisfaction scores as continuous dependent variables, and for items with a dichotomous response (Yes-No), the method of generalized estimating equations (GEE) was used. GEE is an extension of the generalized linear model and allows analysing data with correlated residuals, i.e. clustered data [23, 24]. Data was clustered due to the dyadic design with correlated data between partners (mother and father), based on partner effects and common fate [25]. Diagnostic group was the model’s predictor (factor with four levels) with the neonatology group set as reference for comparison since it was the group with the most cases. To control for potential gender effects independent of the dyadic structure, gender (female/male) was specified as a confounder. For items with multiple choice response options, Pearson’s chi-square or Fisher’s exact test were calculated as appropriate. Contribution to a potential significant main effect was interpreted by breaking down the standardized residuals with values outside ±3.29 representing significance at *P* <0.001 [26]. To adjust for multiple testing, a probability of ≤ 0.001 was set to decide significance. All quantitative data were analysed using IBM® SPSS® Statistics 21 for Mac® (IBM Corp, Armonk, NY, USA).

Text responses from the three open-ended questions concerning support services in bereavement, and positive and negative experiences were imported in the text management program ATLAS.ti7, 7.5.4 for MS Windows® (ATLAS.ti GmbH, Berlin, Germany). These qualitative data were coded deductively, following the questionnaire’s six quality domains, by two trained research assistants independently. The two solutions were merged based on consensus between the two coders. To summarise this information on the item level, frequencies of codes within the domains were counted to demonstrate which domains were prominently presented.

## Results

**Fig. 1:**
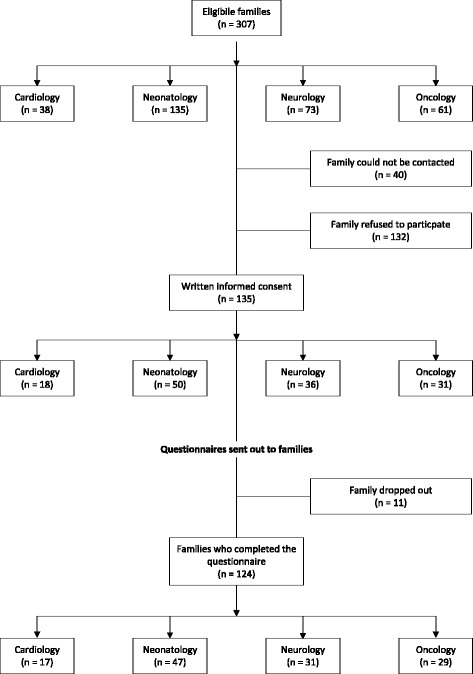
Study recruitment and participation

Flow of study recruitment and participation is displayed in Fig. 1. The participation rate was 51 % (*n* = 135) of the 267 eligible families who received the study documents. Study participation differed between the diagnostic groups with parents from the neonatology group showing the lowest rate and parents from the oncology group the highest (Fig. 1). Of the 224 individual questionnaires (mothers and fathers) sent out to the 135 families, 200 questionnaires from 124 families were completed and sent back, representing a questionnaire response rate of 89 %. Parents of deceased neonates represented the largest group, followed by parents from the neurology, oncology and cardiology group (Table 1). The sample mainly consisted of Swiss residents (87 %), with 13 % migrant families. With neonates comprising the largest group of deceased children, most deaths occurred on a neonatal intensive care unit (ICU), as reported by the parents. The age of the deceased child differed among the diagnostic groups, with neonates obviously being the youngest (Table 1).

**Table 1:** Sample characteristics of parents (*N* = 200), place of death and age of child (*N* = 124)

Characteristics	Cardiology	Neonatology	Neurology	Oncology	Total
	*n* = 26	*n* = 81	*n* = 48	*n* = 45	*N* = 200
	(13 %)	(41 %)	(24 %)	(22 %)	(100 %)
Age^a^, *M* (*SD)*					40 (6.48)
Mothers,					
*n* = 112 (56 %)	38 (4.38)	37 (4.29)	41 (6.07)	43 (7.30)	39 (6.05)
Fathers,					
*n* = 88 (44 %)	40 (6.88)	39 (5.77)	42 (6.56)	48 (5.85)	42 (6.83)
Language, *n* ( %)					
German	21 (80.8)	66 (81.5)	44 (91.7)	31 (68.9)	162 (81.0)
French	5 (19.2)	9 (11.1)	3 (6.3)	12 (26.7)	29 (14.5)
Italian	0 (0.0)	6 (7.4)	1 (2.1)	2 (4.4)	9 (4.5)
Marital status, *n* (%)		*n* = 80			*N* = 199
Married/Partnership	22 (84.6)	79 (98.8)	43 (89.6)	41 (91.1)	185 (93.0)
Divorced/Separated	4 (15.4)	1 (1.3)	4 (8.3)	2 (4.4)	11 (5.5)
Single	0 (0.0)	0 (0.0)	1 (2.1)	2 (4.4)	3 (1.5)
Religious affiliation, *n* (*%*)		*n* = 80		*n* = 44	*N* = 198
Catholic	7 (26.9)	37 (46.3)	21 (43.8)	14 (31.8)	79 (39.9)
Protestant	7 (26.9)	25 (31.3)	17 (35.4)	15 (34.1)	64 (32.3)
None	8 (30.8)	12 (15.0)	3 (6.3)	10 (22.7)	33 (16.7)
Other	4 (15.4)	6 (7.5)	7 (14.6)	5 (11.4)	22 (11.1)
Education, *n* (*%*)					
School levels^b^	0 (0.0)	2 (2.5)	1 (2.1)	5 (11.1)	8 (4.0)
Post school education^c^	11 (42.3)	39 (48.1)	19 (39.6)	20 (44.4)	89 (44.5)
Tertiary level^d^	10 (38.5)	15 (30.9)	22 (45.8)	16 (35.6)	73 (36.5)
University degree	5 (19.2)	15 (18.5)	6 (12.5)	4 (8.9)	30 (15.0)
Employment status at death of the child					
Working	10 (38.5)	41 (50.6)	28 (58.3)	16 (35.6)	95 (47.5)
Off work^e^	16 (61.5)	40 (49.4)	20 (41.7)	29 (64.4)	105 (52.5)
Employment status at time of the survey					
Working	19 (73.1)	65 (80.2)	43 (89.6)	39 (86.7)	166 (83.0)
Off work^e^	7 (26.9)	16 (19.8)	5 (10.4)	6 (13.3)	34 (17.0)
Family income^f^, *n* (*%*)	*n* = 19	*n* = 66	*n* = 44	*n* = 40	*N* = 169
≤ CHF 100,000.-	9 (47.4)	33 (50.0)	17 (38.6)	24 (60.0)	83 (49.1)
> CHF 101,000.-	10 (52.6)	33 (50.0)	27 (61.4)	16 (40.0)	86 (50.9)
Deceased child was the only child, *Yes* (*%*)	1 (3.8)	13 (16.0)	6 (12.5)	4 (8.9)	24 (12.0)
Previous loss of a child, *Yes* (*%*)	4 (15.4)	6 (7.4)	5 (10.4)	2 (4.4)	17 (8.5)
Place of death	*n* = 16	*n* = 51	*n* = 29	*n* = 28	*N* = 124
Intensive care unit	10 (62.5)	47 (92.1)	13 (44.8)	5 (17.8)	75 (60.5)
Hospital	2 (12.5)	0 (0.0)	8 (27.6)	11 (39.3)	21 (16.9)
Home	3 (18.8)	3 (5.9)	6 (20.7)	11 (39.3)	23 (18.6)
Somewhere else	1 (6.2)	1 (2.0)	2 (6.9)	1 (3.6)	5 (4.0)
Deceased child’s age					
in days, *Mdn* (*range*)		5 (1–26)			Na
in years, *Mdn* (*range*)	0.5 (0.1–9.1)		4.8 (0.1–17.2)	8.0 (1.7–17.4)	3.3 (0.1–17.4)

*Note.* Na = not applicable

^a^Age at the time of the survey. ^b^Consists of primary and secondary level. ^c^Consists of college and vocational education. ^d^Consists of degrees from schools of higher education. ^e^Consists of being on sick leave, on unpaid leave, being unemployed or in educational training. ^f^Annual gross pay, Swiss average lies at CHF 143,000.- [45]

True missings over all questionnaire items ranged between 0 and 14 % (socio-demographic items excluded). When the response options “not applicable/don’t know” were counted, the range went from 0 % up to 43 %. Four items were analysed only descriptively because of more than 30 % of missing information.

### Parental experiences and perceived satisfaction with care

**Fig. 2:**
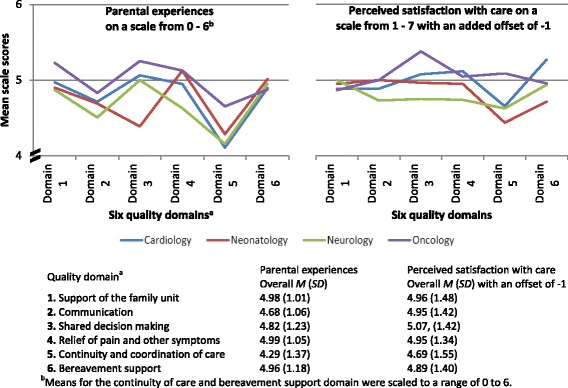
Parental experiences and perceived satisfaction with care according to the six quality domains

Overall parental experiences and their perceived satisfaction with care their child received will first be summarised, followed by more detailed reporting, focusing on differences between the diagnostic groups within the six quality domains. Parents rated experiences with their child’s EOL care as generally positive (Fig. 2). After accounting for the different scoring ranges among the six quality domain scales (7-point and 5-point), experience scores were highest for the domain *relief of pain and other symptoms* (*M* = 4.99, *SD =* 1.05) and lowest for the domain *continuity and coordination of care* (*M =* 4.29, *SD =* 1.37). Across all six domains, parents of children with cancer rated their experiences during their child’s EOL care highest (*M =* 4.80, *SD =* 0.51), while parents of children with a neurological condition rated their overall experiences lowest (*M =* 4.51, *SD =* 0.44). The cardiology, neurology and oncology groups all showed the same pattern in experience scores across the six quality domains. Parents of neonates showed a different pattern, with a significantly lower score in the domain *shared decision making* (main effect, *P* = 0.001) and a high score in the domain *relief of pain and other symptoms* (*M =* 5.13, *SD =* 1.01).

Overall perceived satisfaction with the child’s EOL care was also rated highly, with a mean of 5.92 (*SD* = 1.05) out of 7 across all quality domains and all diagnostic groups. However, the score patterns of the four diagnostic groups across the domains showed a different picture than for experiences. The domain *shared decision making* received the highest satisfaction rating (*M =* 6.07, *SD =* 1.42), and the domain *continuity and coordination of care* the lowest (*M =* 5.69, *SD =* 1.55). Consistently with parental experiences, parents from the oncology group rated their overall perceived satisfaction highest among all groups, while parents from the neurology group rated it lowest (Fig. 2).

#### Support of the family unit

Parents or their dying child had access to a variety of support services. The most frequently reported were pastoral care (*n* = 108 Yes responses), followed by psychological care (*n* = 88) and physiotherapy (provided to the child, *n* = 71). Community, social and bereavement services were less common, but still offered to a quarter of the parents. Again, there were some differences between the diagnostic groups. Pastoral care and bereavement services were predominantly offered to parents of neonates (main effects, *P* = <0.008 and *P* = <0.007 respectively), and physiotherapy to children with a neurological condition (*P* = <0.001). Access to complementary medicine was mostly reported by parents with a child with cancer and almost never by parents of neonates. (*P* = <0.001). Thirty-five (18 %) parents reported that they received specialised palliative care services. This was most often the case for parents of children with cancer (*n* = 7 Yes responses, 38 %), and rarest for parents of neonates (6, 7 %).

#### Communication in general and with physicians

Experiences with the clarity and honesty of the information physicians provided were analysed as single items and are summarised in Table 2. Parents from the oncology group consistently reported the most positive experiences (Table 2). All but seven parents (97 %, *n* = 193) confirmed that they were informed that their child could die. However this occurred at differing time points, depending on the diagnostic group. In the cardiology and neonatology groups 42 % (*n* = 11) and 36 % (*n* = 28) of parents, respectively, reported being informed that their child would die prenatally (this response option was available for those two groups only). Information was provided within 24 h or a few days prior to the child’s death to an additional 59 % (*n* = 46) of parents of neonates and 20 % (*n* = 9) of parents in the neurology group. Most parents in the neurology and oncology group (76 %, *n* = 35, and 89 %, *n* = 40, respectively), and 42 % (*n* = 11) of parents in the cardiology group were informed that their child was likely to die from a few months to more than six months before her/his death. Only the parents in the neurology and oncology groups were asked who had informed their child that she or he could die. In the neurology group most parents (91 %, *n* = 42) reported that their child could not be informed because of the child’s age or mental state. This was less frequently the case in the oncology group (32 %, *n* = 14). Children with cancer were informed about the possibility of dying by their parents alone (27 %, *n* = 12) or by their parents and a physician (25 %, *n* = 11). A few parents from the oncology group reported that they did not want anyone to talk to their child about dying (7 %, *n* = 3) or that their child did not want to talk about it (5 %, *n* = 2).

**Table 2:** Communication domain: parental experiences related to clarity and honesty of information provided by physicians^a^

	Cardiology	Neonatology	Neurology	Oncology	Total
	*M* (*SD*)	*M* (*SD*)	*M* (*SD*)	*M* (*SD*)	*M* (*SD*)
	*Mdn* (*range*)	*Mdn* (*range*)	*Mdn* (*range*)	*Mdn* (*range*)	*Mdn* (*range*)
In general^b^					
Clarity	5.88 (0.82)	6.00 (1.23)	5.83 (1.33)	6.23 (1.10)	5.99 (1.18)
	6 (4–7)	6 (1–7)	6 (2–7)	6 (1–7)	6 (1–7)
Honesty	6.19 (1.10)	6.31 (1.20)	6.17 (1.26)	6.53 (0.84)	6.31 (1.13)
	7 (4–7)	7 (1–7)	7 (2–7)	7 (3–7)	7 (1–7)
Treatment options to alleviate suffering					
Clarity^c^	6.08 (1.02)	5.96 (1.29)	5.79 (1.03)	6.31 (0.85)	6.02 (1.11)
	6 (4–7)	6 (1–7)	6 (3–7)	7 (4–7)	6 (1–7)
Prospects of life-sustaining measures					
Clarity	6.14 (0.96)	6.11 (1.15)	6.37 (0.95)	6.53 (0.72)	6.27 (1.00)
	6 (4–7)	6 (2–7)	7 (2–7)	7 (4–7)	7 (2–7)
Honesty	6.14 (1.35)	6.32 (1.09)	6.36 (1.23)	6.68 (0.62)	6.39 (1.09)
	7 (2–7)	7 (2–7)	7 (1–7)	7 (5–7)	7 (1–7)

*Note.* No statistically significant differences between the diagnostic groups

^a^Parents were asked to rate their experiences concerning communication with the attending physicians in terms of clarity and honesty. ^b^All items were coded on a scale from 1 to 7. ^c^Honesty response option not provided for this item

#### Shared decision making

Overall, 60 % of parents (*n* = 110) reported that a decision about resuscitating their child had been made. Those decisions were made significantly less often within the neonatology group (42 %, *n* = 33), especially when compared with the neurology group (88 %, *n* = 42; main effect, *P* = <0.001). Parents reported that resuscitation-related decisions were made by the family together with the health care team (HCT) in 39 % of the cases (*n* = 45), by the family alone in 34 % (*n* = 40), or by the HCT alone in 20 % of the cases (*n* = 20). More parents in the neurology group reported that the decision was made by the family alone (52 %, *n* = 22), whereas in the other groups it was commonly made by the family together with the HCT. Only parents of neonates were asked whether the cessation of life-sustaining interventions was discussed. Eighty-three percent (*n* = 63) confirmed that it was discussed and that the decision to end those measures was made by the family together with the HCT in 65 % of cases (*n* = 46).

#### Relief of pain and other symptoms

Of all quality domains, experiences with pain management were rated most positively on a scale from 0 to 6 (*M =* 4.99; *SD* = 1.05), and highest of all by parents from the neonatology and oncology group (*M =* 5.13). Parents from the neurology group rated their experiences lowest (*M* = 4.63, *SD =* 1.17). Those parents were also less satisfied and reported the lowest value of all groups within this quality domain (Fig. 2).

Parents were asked to rank the three symptoms from which their child suffered and that were most stressful to them from a list of more than 10 different symptoms. Problems with breathing was ranked most frequently by parents from the cardiology, neonatology and neurology groups, followed by pain. Parents in the oncology group ranked pain first. Breathing problems and pain were overall the symptoms most frequently ranked among the top three as being most stressful for parents. Other stressful symptoms were different according the diagnostic group. For the cardiology group, agitation and anxiety were frequently ranked in first or second place. Parents of neonates frequently ranked circulatory problems in first and third place among the top three. Parents from the neurology group frequently ranked mucus in the airways and seizures among the top three, and for parents from the oncology group, their child’s fatigue and impaired verbal or nonverbal communication frequently appeared among the top three.

#### Continuity and coordination of care

Experiences related to continuity and coordination of care were rated least positively of all six quality domains by all parents. Between the diagnostic groups, parents of children with cancer rated their experiences highest, with a mean of 4.10 (*SD =* 0.81) on a scale from 1 to 5. This was also reflected in the overall satisfaction rating of that domain (on a scale from 1 to 7), where parents from the oncology group were more satisfied (*M =* 6.09, *SD =* 1.20) than parents from the other groups (*M =* 5.53, *SD =* 1.64) (Fig. 2).

Parents from the cardiology, neurology and oncology group were asked who mainly supported them professionally in the organisation of their child’s care. The most frequent answer for all groups was: a hospital-based physician (36 %, *n* = 37). For the oncology group this was followed by a hospital-based nurse (26 %, *n* = 11), which was less often the case for the neurology group (8 %, *n* = 3). Evenly spread across all diagnostic groups, parents also reported that a community-based nurse supported them in organising care (18 %, *n* = 19) but 19 % (*n* = 20) answered that no one supported them. Main support was provided by a primary care paediatrician (PCP) in 8 % of cases (*n* = 8).

### Bereavement support

Sixty-nine percent (*n* = 137) of the parents stayed in contact with someone from the HCT shortly after their child’s death. This applied most often to parents from the oncology group, with only 16 % (*n* = 7) of parents having no further contact. Overall 65 % (*n* = 130) of the parents reported having a follow-up talk with someone from the HCT. The lowest rate was reported by parents from the neurology group, where it was the case in 50 % (*n* = 23) of respondents. Parents were asked to write down what kind of support services they used or still use during their bereavement. We received written information from 140 parents. Of those, 59 (42 %) parents reported making use of psychological support services, followed by support groups with other bereaved parents (32 %, *n* = 45). Other common answers were related to alternative support services (26 %, *n* = 36) such as kinesiology, art therapy, dream therapy, and spiritual services (24 %, *n* = 34) with a pastor or in a religious community.

### Positive and negative experiences and quality of life

Parents were asked to describe three positive and three negative experiences related to their child’s EOL care. Responses were classified according to the questionnaire’s six quality domains and are summarized with frequencies and a sample quote in Table 3. Both positive and negative experience descriptions were most frequently about the support the family received.

**Table 3:** Positive and negative experiences

	Number of quotes^a^	Sample quote
Positive experiences	180	
Support of the family unit	174	*Our individual needs were always supported. (18:6)* ^b^
Communication	54	*Honesty when informing about our child’s situation. (22:2)*
Shared decision making	8	*Ethics council helped to take the right decision. (35:4)*
Relief of pain and other symptoms	8	*Oxygen support at home, mail order of medication. (101:2)*
Continuity and coordination of care	46	*Reachability day and night (hospital and community care). (17:1)*
Bereavement support	39	*That a lot of time was provided* (by the hospital) *to be with my son after his death. (3:1)*
Negative experiences	165	
Support of the family unit	110	*I felt left alone (75:3)*
Communication	73	*Not having enough information about my child’s situation (61:3)*
Shared decision making	14	*The night doctors did not support and follow our decision to end treatments. (5:7)*
Relief of pain and other symptoms	27	*Pain and shortness of breath. There was a phase when effective medication lagged behind the symptoms. (33:10)*
Continuity and coordination of care	53	*When the physicians and nurses always change. (116:2)*
Bereavement support	51	*No follow up care for us after her death. I needed to find my own psychologist/support group. (97:7)*

^a^Each listing of a positive or negative experience (quote) was potentially coded to more than one domain. ^b^Numbers in parenthesis represent the quote’s identification number

Current quality of life (QoL) was rated high overall, with a mean of 7.19 (*SD* = 2.09) on a scale from 0 to 10. However six parents reported low QoL below the 5^th^ percentile with values between 2.30 and 0. Parents who had lost a child due to cancer rated their QoL lower than parents from the other groups with a mean of 6.55 (*SD* = 2.17) vs. cardiology (*M* = 8.09; *SD* = 1.18), neonatology (*M* = 7.46; *SD* = 1.88), and neurology (*M* = 6.84; *SD* = 2.49). To further investigate this difference and based on the sample’s characteristics, the parents’ age and income was added to the GEE model. In this extended model, income was the most influential predictor of QoL (*P* = 0.002) with higher income predicting higher QoL.

**Fig. 3:**
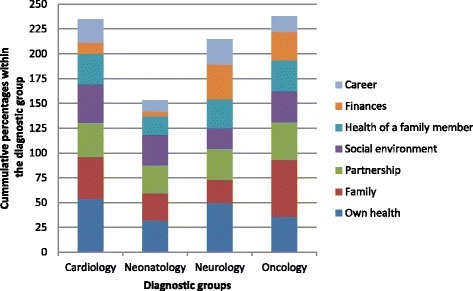
What areas of your personal life were negatively influenced by the illness and death of your child? Parents could choose from a list of seven potential areas and check all that applied. The areas are represented by the different colours. The number of checks per participant was for cardiology 2.3, neonatology 1.5, neurology 2.3, and oncology 2.2

Finances were also one of seven response options for the question about areas of the parents’ personal life that were negatively influenced by the illness and death of their child (Fig. 3). Overall, 37 (18.5 %) parents responded that finances were an area that was negatively influenced. Significantly fewer parents in the neonatal group reported that their finances were negatively affected compared to the other groups (main effect, *P* = <0.001) whereas in parents from the neurology group the proportion of “Yes” responses was highest (35 %). The areas most commonly affected across all diagnostic groups were their own health (*n* = 80, 40 %) followed by the family (*n* = 70, 35 %) and the partnership (*n* = 64, 32 %) (Fig. 3).

## Discussion

This is one of the few studies that quantitatively described and explored parental experiences related to their child’s EOL care in a population-based sample of bereaved mothers and fathers of children from the major diagnostic groups in which childhood deaths occur. This allowed us to compare findings in four distinct diagnostic groups, which adds to existing knowledge about parental perspectives. Overall, parental scores on their experiences and perceived satisfaction with their child’s end of life care were high across all six quality domains. Parents of a child with cancer rated their experiences highest in most of the six quality domains and reported the highest satisfaction with care. The lowest scores were mainly reported by parents from the neurology group, with the exception of the domain *shared decision making*, where parents of neonates reported significantly less positive experiences.

### Satisfaction with care

Evaluation of health care is considered the most important purpose of measuring patient/parental satisfaction [27]. However, ceiling effects are a methodological concern when measuring the construct of satisfaction, i.e. high levels of satisfaction are consistently reported, which reduces the ability to discern differences. Such high levels might be due to inherently low expectations and should thus not be automatically interpreted to mean that care was good but simply that nothing bad happened [27]. This was, for example, the case in Wolfe et al.’s study [28] which showed substantial parent-reported EOL suffering in children with cancer as well as, simultaneously, high levels of satisfaction with care. A similar mechanism might contribute to this study’s high perceived satisfaction levels, e.g. results for the domain *shared-decision making* of the neonatology group. Assessing specific experiences with aspects of care shows promise as a means of overcoming limitations in general satisfaction measures. This is supported in this study where there were more variable results between the six quality domains and four diagnostic groups, and substantial differences were present in regard to some domains. The role of domains is important as they present a structural framework for good quality care and evaluating experiences offers insight into processes of care that is less biased by expectations than measuring satisfaction of care. The domains chosen for the PaPEQU were established by experts in the field and mostly in accordance with domains established through exploratory factor analysis by Widger et al. [29]. Future research however, should further focus on empirical model development and testing to measure good quality paediatric EOL care [19].

### Communication

Parents consider genuine communication with sincere and honest provision of information to be most central [3], and lacking or poor communication were recurrent themes in the Aschenbrenner et al. [2] review and a recent meta-synthesis by Xafis et al. [30]. Experiences and satisfaction scores related to communication in this study were high. However, extreme negative outliers were present and reflected in the parents’ written comments about their negative experiences, where a lack of or inconsistent provision of information and also insensitive communication in general were predominantly described. Similar complaints were also described in a meta-synthesis on the information needs of parents facing an EOL decision for their child [30]. Even though it might affect only a few parents, these experiences should not be taken lightly as their negative impact can last for years after the traumatic event.

### Shared decision making

Consistent with our findings, a majority of paediatric deaths occur in the ICU [31], and, especially for neonates, these are preceded by a decision to limit or withdraw life-sustaining measures [32]. More than 80 % of parents from the neonatal group in this study reported that the withdrawal of life-sustaining treatment had been discussed; however only 65 % indicated that they made those decisions in consultation with the HCT. Some parents felt that the decision was made by them alone or by the HCT alone. This finding contradicts what was found in a another Swiss study evaluating how EOL decisions were made in neonatology and how consistently a framework for structured ethical decision making was applied [33]. They concluded that 92 % of parents were actively involved in the decision by having received full information about the baby’s condition, prognosis, therapeutic possibilities and the approach the treating team would take [33]. A Canadian study exploring processes of death in neonates reported that there was agreement between physicians and parents in 84 % of cases where a decision to withdraw life-sustaining measures was made [32]. All parents in our study were also asked if there had been a decision concerning the potential need to resuscitate their child; 60 % said there had been. However, one third of those parents, predominantly parents from the neurology group, indicated that they perceived themselves making this decision without input from the HCT. Such results are unexpected and call into question our perceived reality of family-centred care. The child’s best interest is always central in making these decisions. However, professional caregivers and parents have their own personal perceptions, values and interpretations of what is best for the child and the balance of power is not equal in this context. Following the traditional principles of bioethics may not ensure that parents have the opportunity to participate to their satisfaction in those important decisions. It might be that other communication models ensuring shared decision making should be considered and introduced. One such model, communicative ethics, is explained and discussed in the context of neonatal intensive care by Daboval, Shidler [34]. It builds on the shared-decision model which is considered as gold standard [35], acknowledging that the decision made cannot be separated by the communicational process used to reach it [34].

### Relief of pain and other symptoms

Although experiences with alleviation of suffering were rated highest among the six quality domains and perceived satisfaction levels second highest, parents still reported that their child experienced a wide variety of stressful symptoms, indicating that significant symptom burden is present at EOL [36]. Breathing changes are part of the dying process and were probably witnessed by most parents. Even though it is recommended that parents be informed about what physical changes to expect when their child is dying, witnessing this process remains very stressful. In a US study with 50 bereaved parents of children with cardiac diagnoses, breathing difficulties were associated with considerable suffering in 77 % of the 30 parents who reported that symptom [15]. Pain was another frequently reported symptom in our study and experiences related to pain management were rated lowest by parents from the neurology group. This reflects the tremendous challenges we face when caring for nonverbal children with a variety of neurological impairments [37].

### Continuity and coordination of care

Continuity and coordination of care is recognized as an important factor in promoting caring, reducing parental frustration, and enhancing parents’ confidence in the quality of their children’s care [38]. Experiences as well as perceived satisfaction with this aspect of care were rated lowest of all domains by parents in this study and point to an area with a need for substantial improvement. Supported transition between inpatient, outpatient and home care is essential to high quality EOL care [4]. This however, requires appropriate structures concerning health care services and professional palliative care support. While parents in our study felt most supported by a hospital-based physician or nurse, many felt left alone. Community-based nurses and PCPs only played a minor role. The latter was also described in a study that explored the involvement of PCPs when their patients faced the EOL [39]. The fact that multidisciplinary PPC teams only exist in three Swiss paediatric hospitals and only 18 % percent of all parents reported having received specialised PPC services might contribute to our study’s findings.

### Bereavement support

Continuity and attention remain important after the death of the child. Parents described great appreciation for staff sending cards or attending the child’s funeral and said that the loss of this connection added to their bereavement [38]. Interpreting our study’s findings, this might be especially true for parents of a child with cancer who, although the palliative care period tends to be rather short, experience a long illness/treatment phase with many hospital stays in a dedicated paediatric oncology unit, creating a special bond between the family and the HCT [40]. Only half of our parents from the neurology group reported having had a follow-up consultation with the former treating team/physician. We cannot conclude whether this reflects those parents’ wishes or rather highlights an area for improvement. Several other studies, however, indicate that parents value the HCT following up with them, that they need help in preparing for what to expect at the time of death, including funeral arrangements, and that they want bereavement services to be available immediately after their child’s death [4]. As also reported by the parents in our study, many desire contact and peer support from other families who have lost a child [40].

### Positive and negative experiences and quality of life

The death of a child disrupts the parents’ well-being and can influence various areas of their personal lives and their quality of life (QoL) [41]. The perception of the quality of medical care has been described as a factor associated with psychosocial morbidity in parents who have lost a child due to cancer [42]. The parents from the oncology group in this study reported the most positive experiences and the highest perceived satisfaction with care. The finding that they reported the lowest current QoL of diagnostic groups was therefore surprising. A similar result was found by Bergstraesser et al. [43] in their study of dyadic coping of parents, where mothers from the oncology group had poorer psychosocial health than fathers and parents from other diagnostic groups. Further exploration of our results revealed that income appeared to be the most influential predictor of QoL. This could in part explain the aforementioned finding, since parents from the oncology group were more frequently in the lower income category. Interestingly, financial strain was described as a major burden for families of children with life-threatening illnesses in Western Australia [14]. The study reported that families in the non-cancer group experienced a high degree of financial strain, which is congruent with our findings that the neurology group reported finances as an area negatively influenced more often than did parents from the other diagnostic groups. Although expenses in this patient group are mostly covered by the Swiss Federal Invalidity Insurance, refund processes seem to work very slowly and putting parents in an economical burdening situation. In addition, financials strains in these families are also due to the duration of care and the fact that one parent will be fully absorbed by the task of care, leading to loss of earning.

### Limitations

Although a high percentage of parents completed the questionnaire, generalisability might still be limited as only parents who had previously provided informed consent received the questionnaire. Our participation rate of 51 % lies within a wide participation range from below 20 % to up to 80 % reported in other studies with bereaved parents [1, 29, 44]. Our findings might be biased in that only parents with favourable experiences may have been motivated to participate. Also, the requirement of being proficient in German, French or Italian excluded some migrant residents representing cultural minorities. The retrospective nature of this study could have introduced a recall bias and parental perceptions of care could have changed over time. However, during the cognitive testing phase of questionnaire development, remembering details of the devastating experience of losing a child was not a problem for participants [19].

## Conclusions

Our findings show positive parental experiences of their child’s EOL care and high perceived satisfaction with the care their child received. In the context of this national study with heterogeneous inpatient and community care settings the differences between the four diagnostic groups were small and within one scoring category. Nevertheless there are some areas worthy of our attention. Parents of neonates reported significantly lower experience ratings related to shared decision-making. As these parents mostly face a decision to withdraw life-sustaining measures, particular attention should be paid to shared decision-making processes. Apparent consensus between the parents and the HCT does not imply that the process was well perceived by the parents. Differences as to how discussions went and on the opportunities or time available might be present.

Parents of children with neurological impairments face many challenges. Symptom management can be a source of distress for parents, as the children are mostly nonverbal and the potential for suffering is high due to a variety of impairments. This makes them dependent on a variety of different care services, which creates a highly complex care environment with a great need for continuity and coordination. Experiences with continuity and coordination of care were rated lowest of all quality domains by parents from all diagnostic groups, and perceived satisfaction with care within this domain was lowest as well. This might be the direct result of lacking specialised PPC services. We have to recognize that the integration of specialised PPC has the potential to minimise lost opportunities for supporting and assisting parents. This has been acknowledged by policy in many countries and there is a growing availability of specialised PPC programs worldwide; unfortunately Switzerland lags behind in this area.

However, the development and implementation of needs-driven and specialised services will fall behind if the benefits of these services are not evidenced in the near future. Structural evaluation and performance data provide one part of the evaluation. However intervention research is needed evaluating processes and outcomes that are meaningful to patients and their families, siblings included. Promoting the best possible outcomes after such a devastating experience has implications for the whole family, the healthcare system and society.

## Additional files

Additional file 1:
**List of all Human Research Ethics Committees that approved the PELICAN study.** (PDF 106 kb) Additional file 2:
**Complete list of items of the PaPEQu with corresponding response options.** (PDF 92 kb)

## Abbreviations

EOL: end-of-life
GEE: generalized estimating equation
HCT: health care team
ICU: intensive care unit
PaPEQu: parental PELICAN questionnaire
PC: palliative care
PCP: primary care paediatrician
PELICAN: paediatric end-of-life care needs
PPC: paediatric palliative care
QoL: quality of life
