# Erratum to: When parents face the death of their child: a nationwide cross-sectional survey of parental perspectives on their child’s end-of life care

**DOI:** 10.1186/s12904-017-0188-x

**Published:** 2017-02-17

**Authors:** Karin Zimmermann, Eva Bergstraesser, Sandra Engberg, Anne-Sylvie Ramelet, Katrin Marfurt-Russenberger, Nicolas Von der Weid, Chantal Grandjean, Patricia Fahrni-Nater, Eva Cignacco, on behalf of the PELICAN Consortium

**Affiliations:** 1 Institute of Nursing Science, University of Basel, Bernoullistrasse 28, 4056 Basel, Switzerland; 2 Department of Paediatrics, Inselspital Bern University Hospital, Bern, Switzerland; 3 Paediatric Palliative Care, University Children’s Hospital Zurich, Steinwiesstrasse 75, 8032 Zurich, Switzerland; 4 School of Nursing, University of Pittsburgh, 3500 Victoria Street, Pittsburgh, PA 15261 USA; 5 Institute of Higher Education and Research in Healthcare - IUFRS, University of Lausanne, Route de la Corniche 10, 1010 Lausanne, Switzerland; 6 Paediatric Department, Lausanne University Hospital CHUV, Rue du Bugnon 21, 1011 Lausanne, Switzerland; 7 Department of Paediatric Haematology-Oncology, University Children’s Hospital UKBB, Spitalstrasse 33, 4056 Basel, Switzerland; 8 Paediatric Palliative Care Team, Lausanne University Hospital CHUV, Rue du Bugnon 21, 1011 Lausanne, Switzerland; 9 Research in Midwifery, University of Applied Sciences Bern, Health Division, Bern, Switzerland

## Erratum

During production of the original article [1], Additional file 2 of this article was linked to an incorrect document. Instead of the complete item list of the PaPEQu as the authors submitted it, it was a STROBE checklist. The correct additional file has been included with this erratum.

## Additional file

Additional file 2: Complete list of items of the PaPEQu with corresponding response options. (PDF 200 kb)
