# Supplementary material for: When parents face the death of their child: a nationwide cross-sectional survey of parental perspectives on their child’s end-of life care
Source: BMC Palliat Care. 2016 Mar 9;15:30. doi: 10.1186/s12904-016-0098-3 (PMC4784404; doi:10.1186/s12904-016-0098-3)
Supplement: Additional file 1: — List of all Human Research Ethics Committees that approved the PELICAN study. (PDF 106 kb) [file 12904_2016_98_MOESM1_ESM.pdf]

List of all Human Research Ethics Committees that approved the PELICAN study

Swiss canton	Committee's full name	Study reference number	Date of approval
Zurich	**Leitethikkommission:** Kantonale Ethik-Kommission Zürich (KEK)	KEK-ZH-Nr. 2012-0537	11. 03. 2013
Aargau	Kantonale Ethikkommission Aargau	2013/030	21.05.2013
Basel	Ethikkommission beider Basel	101/13	23.05.2013
Bern	Kantonale Ethikkommission Bern (KEK)	KEK-BE: 058/13	07.05.2013
Genève	Commission d'éthique de la recherche sur l'être humain (CEREH)	CER: 13-150	23.07.2013
Luzern	Kanton Luzern, Gesundheits- und Sozialdepartement, Dienststelle Gesundheit, Ethikkommission	Keine	02.05.2013
St. Gallen	Kanton St. Gallen, Ethikkommission	EKSG 13/042	06.05.2013
Ticino	Comitato etico cantonale	CEL 2704	01.07.2013
Thurgau	Kantonale Ethikkommission Thurgau	2013/21	08.08.2013
Vaud	Commission cantonale d'éthique de la recherché sur l'être humain	NE230/13	30.08.2013
Valais	Commission cantonale valaisanne d'éthique médicale	CCVEM 024/13	17.06.2013
