# Supplementary material for: Erratum to: When parents face the death of their child: a nationwide cross-sectional survey of parental perspectives on their child’s end-of life care
Source: BMC Palliat Care. 2017 Feb 17;16:16. doi: 10.1186/s12904-017-0188-x (PMC5314625; doi:10.1186/s12904-017-0188-x)
Supplement: Additional file 2: — Complete list of items of the PaPEQu with corresponding response options. (PDF 200 kb) [file 12904_2017_188_MOESM1_ESM.pdf]

List of items of all PaPEQu versions*

**Support of the family unit domain**	**Presence in questionnaire version**			
**Items assessing parental experience:** *7 point adjectival scale, never – always*	**Card**	**Neo**	**Neur**	**Onc**
(1) I felt taken seriously with the difficult situation my child and I were in.	✓	✓	✓	✓
(2) I could be involved in my child's care as much as I wanted to be.	✓	✓	✓	✓
(3) a. When I needed respite there was a professional (e.g. nurse, volunteer) who could take my place.	✓	✓	✓	✓
(3) b. My privacy was respected.	✓	✓	✓	✓
(4) The care team was aware of my worries and fears.	✓	✓	✓	✓
(5) I trusted the healthcare professionals.	✓	✓	✓	✓
(6) What services were offered to you or your child during the child's last four weeks of life? *List of 10 possible services to choose all those applicable + Other.*	✓	✓	✓	✓
**Items assessing parental needs:** *7 point adjectival scale, not important at all – very important*				
I needed				
(7) a. To have a place to sleep in the hospital close to my child. Response option *"not applicable"* available	✓	✓		
(7) b. To be involved in my child's care.			✓	✓
(8) a. To have a room where my family and I could spend some private time together.	✓	✓		
(8) b. To have respite from the care of my child.			✓	✓
(9) To share my fears and worries with someone from the healthcare team.	✓	✓	✓	✓

**Communication domain**	**Presence in questionnaire version**			
**Items assessing parental experiences:** *7 point adjectival scale, never – always*	**Card**	**Neo**	**Neur**	**Onc**
(10) Information concerning my child's condition was provided appropriately.	✓	✓	✓	✓
(11) Bad news was communicated in a sensitive manner.	✓	✓	✓	✓
(12) Information I received was contradictory.	✓	✓	✓	✓
(13) My questions were taken seriously by the healthcare team.	✓	✓	✓	✓
(14) To get information concerning my child's condition I had to ask.	✓	✓	✓	✓
(15) I had the impression that the information I received about treatment options was complete.	✓	✓	✓	✓
**How did you experience communication with the attending physicians?** *7 point adjectival scale, not clear at all – very clear*				
(16) In general: Additionally: *7 point adjectival scale, not honest – honest*	✓	✓	✓	✓
(17) About treatment options to alleviate symptoms:	✓	✓	✓	✓

*Card, cardiology; Neo, neonatology; Neur, neurology; Onc, oncology.

(18)	About the prospects and limitations of life-sustaining measures:<br>Additionally: *7 point adjectival scale, not honest – honest*<br>Response option *“not applicable”* available	✓	✓	✓	✓
(19)	About what kind of physical changes to expect when my child is dying:<br>Additionally: *7 point adjectival scale, not honest – honest*<br>Response option *“not applicable”* available	✓	✓	✓	✓
(20)	Were you informed that your child could die?<br>*Yes – No*	✓	✓	✓	✓
(21)	a. When were you informed that your child could die?<br>Multiple choice: *A few days before my child died – Less than 4 weeks before my child died – Less than 6 months before my child died – More than 6 months before my child died*			✓	✓
(21)	b. When were you informed that your child could die?<br>Multiple choice: *Before the birth of my child – Less than 24 hours before my child died – Less than 1 week before my child died – 2 to 4 weeks before my child died*		✓		
(21)	c. When were you informed that your child could die?<br>Multiple choice: *Before the birth of my child – Less than 24 hours before my child died – A few days before my child died – Less than 4 weeks before my child died – Less than 6 months before my child died – More than 6 months before my child died*	✓			
(22)	Who informed your child that she/he could die?<br>Multiple choice: *It was not possible to inform my child – Myself or the other parent – A physician together with me or the other parent – A physician alone – My child did not want to speak about it – I did not want anyone to speak to my child about dying.*			✓	✓
	**Items assessing parental needs:**<br>*7 point adjectival scale, not important at all – very important*				
	I needed				
(23)	To have the opportunity to ask questions at all times.	✓	✓	✓	✓
(24)	To be continuously informed about my child’s condition.	✓	✓	✓	✓
(25)	To find out how my child would die.	✓	✓	✓	✓
(26)	a. To be informed early about my child’s imminent death.	✓	✓		
(26)	b. To be supported in maintaining hope despite the hopeless situation.			✓	✓
	**Shared decision making domain**	**Presence in questionnaire version**			
	**Items assessing parental experiences:**<br>*7 point adjectival scale, never – always*	**Card**	**Neo**	**Neur**	**Onc**
(27)	I was involved in taking decisions.	✓	✓	✓	✓
(28)	I was sufficiently informed to contribute to the decisions taken.	✓	✓	✓	✓
(29)	I had the opportunity to question prior decisions and to re-discuss them.	✓	✓	✓	✓
(30)	Was a decision taken concerning the potential need to resuscitate your child?<br>*Yes – No*	✓	✓	✓	✓

*Card, cardiology; Neo, neonatology; Neur, neurology; Onc, oncology.

(31)	Who decided in favor or against potential resuscitation of your child?<br>Multiple choice: *The topic was never discussed – Me – The other parent – Us as family – The healthcare team – My family together with the healthcare team – Someone else*	✓	✓	✓	✓
(32)	a. Was the cessation of non-helpful treatments discussed with you?<br>*Yes – No*			✓	✓
(32)	b. Was the cessation of life-sustaining measures discussed with you?<br>Yes – No		✓		
(33)	a. Who decided in favour of or against the cessation of non-helpful treatments?<br>Multiple choice: *The topic was never discussed – Me – The other parent – Us as family – The healthcare team – My family together with the healthcare team – Someone else*			✓	✓
(33)	b. Who decided in favour of or against the cessation of life-sustaining measures?<br>Multiple choice: *The topic was never discussed – Me – The other parent – Us as family – The healthcare team – My family together with the healthcare team – Someone else*		✓		
(34)	Did you receive written documentation concerning these decisions?<br>*Yes – No*			✓	✓
	**Items assessing parental needs:**<br>*7 point adjectival scale, not important at all – very important*				
I needed					
(35)	To be involved in taking decisions.	✓	✓	✓	✓
(36)	That my personal beliefs and values were considered when taking decisions.	✓	✓	✓	✓
(37)	Not to have the feeling that I had to take decisions all by myself.	✓	✓	✓	✓
(38)	a. That the cessation of life-sustaining measures was discussed with me.		✓		
(38)	b. That the measures to resuscitate my child were discussed with me.	✓			
(38)	c. That the cessation of non-helpful treatments was discussed with me			✓	✓

**Relief of pain and other symptoms domain**		**Presence in questionnaire version**			
	**Items assessing parental experiences:**<br>*7 point adjectival scale, never – always*	**Card**	**Neo**	**Neur**	**Onc**
(39)	It was my impression that my child's discomforts were eased in the best way possible.	✓	✓	✓	✓
(40)	My child's pain was recognized.	✓	✓	✓	✓
(41)	My child's pain was adequately treated.	✓	✓	✓	✓
(42)	Which of the following 3 of your child's discomforts stressed you the most?<br>List of 12 to 17 different symptoms of discomfort to choose from and rate in descending order.	✓	✓	✓	✓

*Card, cardiology; Neo, neonatology; Neur, neurology; Onc, oncology.

	**Items assessing parental needs:**<br>*7 point adjectival scale, not important at all – very important*				
I needed					
(43)	That my child received enough medication to ease her/his suffering.	✓		✓	✓
(44)	To have physical contact with my child.		✓		
(45)	That my child was awake and receptive enough to be able to play/speak/or do things with us or other people around.			✓	✓
(46)	That my child received medication to calm her/him.		✓		
(47)	That my child received complementary and alternative medicine.	✓		✓	✓
(48)	To be able to use non-pharmacological measures to ease my child's suffering, e.g. massage, tucking.		✓		
(49)	To take my child in my arms.	✓			
(50)	That my child received fluids until the end.	✓		✓	✓
(51)	That I could give my child milk, either through the tube, with a bottle or a cotton swab.		✓		
**Continuity and coordination of care domain**		**Presence in questionnaire version**			
	**Items assessing parental experiences:**<br>*5 point Likert scale, strongly disagree - rather disagree – agree partly – rather agree – strongly agree*<br>Response option *"not applicable"* available	**Card**	**Neo**	**Neur**	**Onc**
(52)	There was a healthcare professional who coordinated my child's care.	✓	✓	✓	✓
(53)	In the hospital, there was a physician in charge of our case whom I could always talk to.	✓	✓	✓	✓
(54)	a. In the hospital, there was a nurse in charge of our case whom I could always talk to.	✓	✓		
(54)	b. At home, my child's care was mostly provided by the same nurse.			✓	✓
(55)	Information concerning the condition of my child was appropriately shared among the whole healthcare team.	✓	✓	✓	✓
(56)	Who mainly supported you in the organization of your child's care during her/his last four weeks of life?<br>Multiple choice: *List of 10 possible professionals and combinations to choose the most applicable*	✓		✓	✓
	**Items assessing parental needs:**<br>*7 point adjectival scale, not important at all – very important*				
I needed					
(57)	To have a professional from the healthcare team to coordinate the care of my child.	✓	✓	✓	✓
(58)	To have the same physician providing care.	✓	✓	✓	✓
(59)	That my child's care was mostly provided by the same nurses.	✓	✓	✓	✓

*Card, cardiology; Neo, neonatology; Neur, neurology; Onc, oncology.

Bereavement support domain		Presence in questionnaire version			
	**Items assessing parental experiences:**<br>*5 point Likert scale, strongly disagree - rather disagree – agree partly – rather agree – strongly agree*<br>Response option *“don’t know”* available	**Card**	**Neo**	**Neur**	**Onc**
(60)	I could spend as much time with my dying child as I wanted to.	✓	✓	✓	✓
(61)	a. I received the necessary support from the healthcare team so that my child could die where I wished.	✓		✓	✓
(61)	b. I was supported by the healthcare team in creating mementos of my child.		✓		

*Card, cardiology; Neo, neonatology; Neur, neurology; Onc, oncology.

		Card	Neo	Neur	Onc
(62)	I could say goodbye to my child in the way I wanted to.	✓	✓	✓	✓
(63)	There was solace around my child’s death	✓	✓	✓	✓
(64)	a. Where did your child pass away?<br>Multiple choice: *In the paediatric intensive care unit – In the adult intensive care unit – On a ward in the paediatric hospital – On a ward in an adult hospital – At home – At another place*	✓		✓	✓
(64)	b. Where did your child pass away?<br>Multiple choice: *In the neonatal intensive care unit - In the paediatric intensive care unit - On a ward in the paediatric hospital - At home – At another place*		✓		
(65)	Were you with your child when she/he passed away?<br>*Yes - No*	✓	✓	✓	✓
(66)	Were siblings, other family members and/or friends with your child when she/he passed away?<br>*Yes - No*	✓	✓	✓	✓
(67)	Did you wash and dress your child or help others to do it after she/he passed away?<br>*Yes - No*	✓	✓	✓	✓
(68)	Were you in contact with someone from the healthcare team during the first weeks after the death of your child?<br>*Yes - No*	✓	✓	✓	✓
(69)	Has there been a follow-up meeting with someone from the healthcare team?<br>*Yes - No*	✓	✓	✓	✓
(70)	Could you please tell us what kind of support services you used or still use during your bereavement?<br>*Free text field to describe the kind of service(s).*	✓	✓	✓	✓
	**Items assessing parental needs:**<br>*7 point adjectival scale, not important at all – very important*				
I needed					
(71)	To have the choice of where child might die.<br>Response option *“not applicable”* available	✓	✓	✓	✓
(72)	That family and friends could say goodbye to my child.	✓	✓	✓	✓
(73)	That I was supported by the healthcare team to structure the hours after the death of my child according my needs.	✓	✓	✓	✓
(74)	To take my child home after her/his death so that family and friends could say goodbye.<br>Response option *“not applicable”* available	✓	✓	✓	✓

*Card, cardiology; Neo, neonatology; Neur, neurology; Onc, oncology.

		Card	Neo	Neur	Onc
(75)	That someone from the healthcare team attended my child's funeral or burial.<br>Response option *"not applicable"* available	✓	✓	✓	✓
(76)	To stay in contact with someone from the healthcare team after my child's death.	✓	✓	✓	✓
**Summary**		**Presence in questionnaire version**			
	**Overall, how satisfied were you with the:**<br>*7 point adjectival scale, not satisfied at all – neutral - totally satisfied*	**Card**	**Neo**	**Neur**	**Onc**
(77)	Support for you and your family?	✓	✓	✓	✓
(78)	Communication with you and your family?	✓	✓	✓	✓
(79)	Shared decision-making?	✓	✓	✓	✓
(80)	Relief of pain and other symptoms?	✓	✓	✓	✓

*Card, cardiology; Neo, neonatology; Neur, neurology; Onc, oncology.

		Card	Neo	Neur	Onc
(81)	Continuation and coordination of care?	✓	✓	✓	✓
(82)	Bereavement support	✓	✓	✓	✓
(83)	Could you please list three positive experiences concerning the care you and your family received:<br>*Free text field to list up to three examples.*	✓	✓	✓	✓
(84)	Could you please list three negative experiences concerning the care you and your family received:<br>*Free text field to list up to three examples*	✓	✓	✓	✓
(85)	What areas of your personal life were negatively influenced by the illness and death of your child?<br>*List of 7 potential areas to choose from; choose all those applicable + Other.*	✓	✓	✓	✓
(86)	How would you rate your current quality of life on the following scale?<br>*Vertical visual analogue scale ranging from 0 (worst possible) to 10 (maximum)*.	✓	✓	✓	✓
(87)	Is there something else you would like to tell us?<br>*Free text field to write.*	✓	✓	✓	✓

Note. The 13 socio-demographic items are excluded from this list.

*Card, cardiology; Neo, neonatology; Neur, neurology; Onc, oncology.
